# Genomic Profiling of *KRAS/NRAS/BRAF/PIK3CA* Wild-Type Metastatic Colorectal Cancer Patients Reveals Novel Mutations in Genes Potentially Associated with Resistance to Anti-EGFR Agents

**DOI:** 10.3390/cancers11060859

**Published:** 2019-06-20

**Authors:** Anna Maria Rachiglio, Matilde Lambiase, Francesca Fenizia, Cristin Roma, Claudia Cardone, Alessia Iannaccone, Antonella De Luca, Marianeve Carotenuto, Daniela Frezzetti, Erika Martinelli, Evaristo Maiello, Fortunato Ciardiello, Nicola Normanno

**Affiliations:** 1 Cell Biology and Biotherapy Unit, Istituto Nazionale Tumori-IRCCS-Fondazione G. Pascale, 80131 Naples, Italy; anmarachiglio@yahoo.it (A.M.R.); matilde.lambiase@libero.it (M.L.); francesca.fenizia@hotmail.it (F.F.); cristin.roma@gmail.com (C.R.); alessia.iannaccone@hotmail.it (A.I.); antoneldel@hotmail.com (A.D.L.); mn.carotenuto@gmail.com (M.C.); daniela_frezzetti@yahoo.it (D.F.); 2 Department of Precision Medicine, Università degli Studi della Campania L Vanvitelli, 80131 Naples, Italy; claudia.cardone88@gmail.com (C.C.); erikamartinelli75@yahoo.it (E.M.); fortunatociardiello@yahoo.com (F.C.); 3 Department of food and feed control, Istituto Zooprofilattico Sperimentale del Mezzogiorno, 80055 Portici (NA), Italy; 4 Department of Oncology, IRCCS Casa Sollievo della Sofferenza, 71013 San Giovanni Rotondo (FG), Italy; e.maiello@libero.it

**Keywords:** colorectal cancer, anti-EGFR monoclonal antibodies, resistance, genomic profiling

## Abstract

Previous findings suggest that metastatic colorectal carcinoma (mCRC) patients with *KRAS/NRAS/BRAF/PIK3CA* wild-type (quadruple-wt) tumors are highly sensitive to anti-epidermal growth factor receptor (EGFR) monoclonal antibodies (MoAbs). However, additional molecular alterations might be involved in the de novo resistance to these drugs. We performed a comprehensive molecular profiling of 21 quadruple-wt tumors from mCRC patients enrolled in the “Cetuximab After Progression in *KRAS* wild-type colorectal cancer patients” (CAPRI-GOIM) trial of first line FOLFIRI plus cetuximab. Tumor samples were analyzed with a targeted sequencing panel covering single nucleotide variants (SNVs), insertions/deletions (Indels), copy number variations (CNVs), and gene fusions in 143 cancer-related genes. The analysis revealed in all 21 patients the presence of at least one SNV/Indel and in 10/21 cases (48%) the presence of at least one CNV. Furthermore, 17/21 (81%) patients had co-existing SNVs/Indels in different genes. Quadruple-wt mCRC from patients with the shorter progression free survival (PFS) were enriched with peculiar genetic alterations in *KRAS*, *FBXW7*, *MAP2K1*, and *NF1* genes as compared with patients with longer PFS. These data suggest that a wide genetic profiling of quadruple-wt mCRC patients might help to identify novel markers of de novo resistance to anti-EGFR MoAbs.

## 1. Introduction

The median overall survival (mOS) of patients affected by metastatic colorectal carcinoma (mCRC) has notably increased in the past 20 years, from 12 months using 5-fluorouracil-based chemotherapy to around 20–30 months with combination therapies including target-based agents [1]. In particular, agents that block the epidermal growth factor receptor (EGFR), such as the anti-EGFR monoclonal antibodies (MoAbs) cetuximab or panitumumab, are an effective therapeutic option in combination with chemotherapy in mCRC patients [2,3].

Treatment with anti-EGFR agents is currently recommended only for mCRC patients with *KRAS/NRAS/BRAF* wild type (wt) tumors, because mutations in these genes have been shown to determine resistance to anti-EGFR therapies [4,5]. Results from different clinical trials also suggest that anti-EGFR MoAbs significantly improve survival only in patients with tumors in the left colon [6]. However, the difference in outcome between left- and right-sided CRC is likely to reflect the different molecular landscapes of these tumors. Indeed, a number of genetic alterations might play a role in the de novo resistance to anti-EGFR agents in mCRC. In particular, single nucleotide variants (SNVs), copy number variations (CNVs) and/or rearrangements in *PIK3CA*, *PTEN*, *ERBB2*, *MAP2K1*, *NTRK1-3*, *RET*, *AKT1*, *ALK*, and *ROS1*, have been claimed to be associated with resistance to anti-EGFR MoAbs [7,8,9,10,11]. However, most of these observations derive from retrospective analyses of patients treated with anti-EGFR agents in advanced lines of treatment.

The “Cetuximab After Progression in *KRAS* wild-type colorectal cancer patients” (CAPRI) study enrolled *KRAS* exon 2 wt mCRC patients who received first-line FOLFIRI plus cetuximab, and at progression were randomized to FOLFOX alone or FOLFOX plus cetuximab. In first-line, the subgroup of patients with *KRAS/NRAS/BRAF/PIK3CA* wt (quadruple-wt) tumors had a better overall response rate (ORR; 64.4%) and median progression free survival (mPFS; 11.3 months), compared with patients harboring a mutation in any of these genes (ORR 47.4% and mPFS 7.7 months) [12,13].

The CAPRI-GOIM cohort represents a unique collection of tumor samples from mCRC patients treated with first-line anti-EGFR agents within an academic clinical trial. The availability of these tumor samples with annotated clinical data offers the possibility to identify novel genetic alterations that might be associated with de novo resistance to anti-EGFR MoAbs. Starting from the hypothesis that the quadruple-wt cohort might be enriched with rare genetic alterations involved in the sensitivity/resistance to anti-EGFR MoAbs, we performed a comprehensive genomic profiling of a subgroup of quadruple-wt tumors from patients enrolled in the CAPRI trial. By using this approach, we could identify potential candidate genes involved in the resistance to anti-EGFR agents, thus suggesting that selection of mCRC patients for treatment with anti-EGFR monoclonal antibodies can be further optimized.

## 2. Results

### 2.1. Targeted Sequencing of KRAS/NRAS/BRAF/PIK3CA Wt mCRC Samples

In order to identify possible mechanisms of resistance to anti-EGFR MoAbs in CRC, we analyzed tumor samples from 21 *KRAS/NRAS/BRAF/PIK3CA* wt mCRC patients enrolled in the CAPRI-GOIM clinical trial by targeted sequencing (Table S1). In particular, we tested the tumor specimens with the Oncomine Comprehensive Panel that provides information on hotspot mutations of 73 oncogenes, CNVs of 49 genes, full-length sequence of 26 tumor suppressor genes, and sequence of 22 driver gene fusions (see Materials and Methods).

The analysis revealed in all 21 patients the presence of at least 1 mutation and in 10/21 (47.6%) the presence of at least one CNV. Furthermore, 17/21 patients had co-existing genetic alterations in different genes (Table S1).

**Figure 1:**
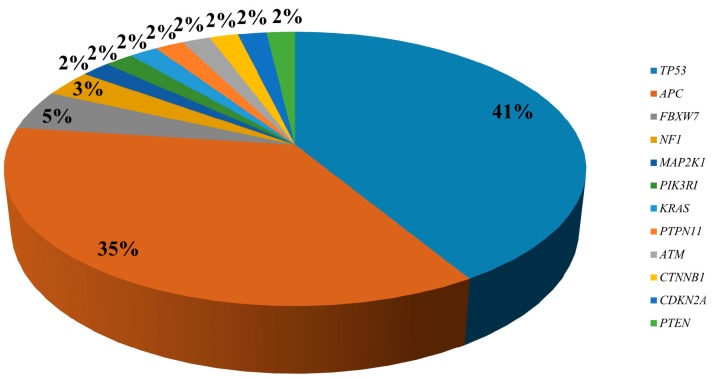
Percentage distribution of the 54 single nucleotide variants (SNVs) and Indels identified in the quadruple-wt mCRC patients.

Of the 54 SNVs and insertions/deletions (Indels) identified, 35% and 41% were *APC* and *TP53* variants, respectively (Figure 1). Nineteen patients (90.47%) had at least one TP53 SNV or Indel, whereas 15/21 (71.43%) patients carried *APC* mutations. All cases with *APC* mutations had also *TP53* variants. Four tumors carried two *APC* variants, one tumor had two *TP53* mutations and one tumor showed three co-existing *TP53* mutations. Two different variants in *TP53* (c.275_276insGGCC and c.837_838InsG) and three in *APC* (c.4467_4468insCATTTTG, c.4098_4099delTCinsAT, and c.589_590insGAGTT) have not been reported in any other sample in public databases to date (www.cbioportal.org; http://cancer.sanger.ac.uk/ cosmic, last accessed 03/14/2019). Mutations were also detected in *FBXW7* (n. 3), *NF1* (n. 2), *MAP2K1* (n. 1), *KRAS* (n. 1), *PTPN11* (n. 1), *ATM* (n. 1), *CTNNB1* (n. 1), *PIK3R1* (n. 1), *PTEN* (n. 1), and *CDKN2A* (n. 1). All genetic variants were confirmed by Sanger sequencing or droplet digital PCR (ddPCR). The relative frequency of the SNVs/Indels is shown in Figure 1.

**Figure 2:**
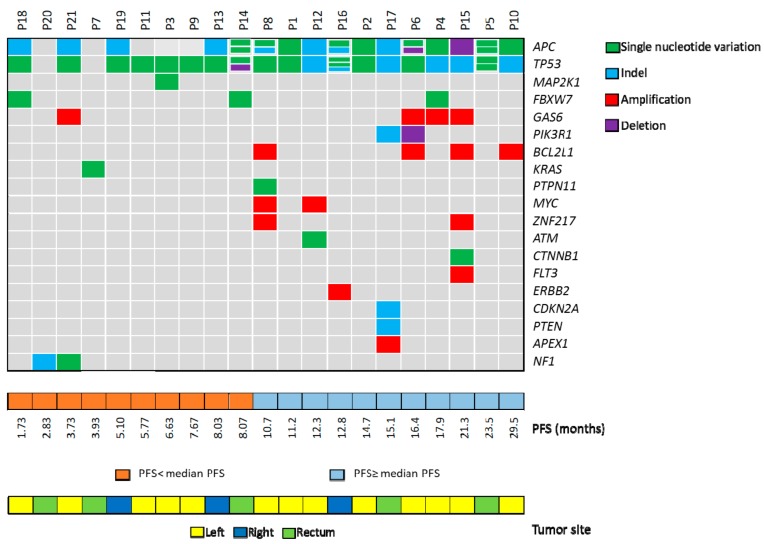
Molecular profile of quadruple-wt mCRC tumors. single nucleotide variants (SNVs), Indels and copy number variations (CNVs) of mutated genes for each patient are represented. Patients’ IDs are shown at the top. Green rectangles represent SNVs. Light blue rectangles represent Indels. Red rectangles represent amplifications. Purple rectangles represent deletions. In the lower panels, progression free survival (PFS) and tumor localization of our cohort of patients are displayed.

The presence of at least one CNV in APC, *TP53, PIK3R1, BCL2L1, GAS6, MYC, ZNF217, FLT3, ERBB2*, and *APEX1* was observed in 10/21 (47.6%) cases. In particular, one case showed deletions of both *APC* and *PIK3R1* (P6), two had deletions of either *APC* (P15) or *TP53* (P14). The other genes showed copy number gains ranging between 4.67 and 78.99. Three tumors (30%) had several amplified or deleted genes (Table S1 and Figure 2).

No gene fusions were detected in 19 tumors. The analysis failed in two cases (P9 and P18).

### 2.2. Correlation of Genetic Landascape with Patients’ Outcome

The median PFS of the 21 patients included in this analysis was 10.7 months (95% CI 6.25–14.87) and the median OS was 32.6 months (95% CI 24.97–41.28), comparable to those observed in the whole cohort of quadruple-wt patients of the CAPRI-GOIM trial [12].

Interestingly, we observed that patients with the shorter PFS (<median PFS) had peculiar genetic alterations in genes involved in the RAS/MEK and mTOR pathways that might be associated with resistance to anti-EGFR drugs. In contrast, only one *FBXW7* variant was observed among patients with a PFS ≥ median PFS (Figure 2).

All variants were at an allelic frequency >5% with the exception of a *KRAS* variant (c.183A>T; p.Gln61His) that was identified in the tumor tissue from patient P7 (PFS 3.93 months) at an allelic frequency of 0.4%. This variant was at an allelic frequency below the 2% sensitivity of the targeted sequencing panel used for tumor molecular profiling in the CAPRI-GOIM trial [12]. The *KRAS* mutation was confirmed by ddPCR analysis of tumor tissue. In addition, the same variant was detected with BEAMing in the cell-free DNA (cfDNA) from the same patient, thus confirming the specificity of the NGS analysis (data not shown).

Patient P3 (PFS 6.63 months) carried the variant c.169A>G in the *MAP2K1* gene coding for the MEK1 protein. This variant has been already reported in the cBioPortal database. It results in the substitution of an amino acid residue (p.Lys57Glu) in the MEK1 negative regulatory domain and it has been associated with a gain of function of the protein [14,15].

Two patients (P20 and P21) had variants in *NF1*, a negative regulator of RAS, inactivated by mutation in various cancers. Specifically, we found an insertion (c.638_639insA; p.Asn214Lys fs*2) in the tumor from patient P20 (PFS 2.83 months) and a SNV (c.5101A>T; p.Lys1701Ter) in the tumor from patient P21 (PFS 3.73 months). These mutations have not been reported in both COSMIC and cBioPortal databases. Both *NF1* mutations lead to the formation of a premature stop codon with consequent loss of function and increased activation of the RAS signaling pathway. Patient P21 carried also a *GAS6* amplification (copy number gain 6.04).

Of the three missense mutations detected in *FBXW7*, two were found in patients with a PFS shorter than median PFS. Patient P14 (PFS 8.07 months) carried the c.1798G>A variant (p.Asp600Asn) and patient P18 (PFS 1.73 months) the c.1513C>T SNV (p.Arg505Cys). The biological effect of the variant p.Asp600Asn is still unknown. In contrast, the FBXW7 p.Arg505Cys mutation has been reported in several cancer types and leads to loss of function of the protein [16].

In 4/10 cases with a PFS <median PFS no genetic alterations in addition to *TP53* and/or *APC* mutations were found. Two of these cases were right-sided colon tumors.

The CNVs were much more frequent among patients with longer PFS. Within this cohort, patient P16 had a significant copy number gain of *ERBB2* (78.99) that was confirmed by FISH analysis (data not shown). Patient P16 had a partial response to cetuximab-based first-line therapy with a PFS of 12.8 months. Patient P4 (PFS 17.93 months), carrying both *GAS6* copy gain (5.59) and a *FBXW7* variant (c.1268G>T; p.Gly423Val), had a complete response to first-line therapy.

The tumor from patient P17 carried several genetic alterations, including an Indel in *PTEN*. Loss of PTEN expression has been previously associated with resistance to anti-EGFR MoAbs [17,18,19]. However, this patient had a first-line PFS of 15.1 months.

### 2.3. Frequency of Identified Genetic Alterations in CRC

Although we identified *MAP2K1*, *FBXW7*, and *NF1* variants in patients with relatively short PFS, this finding could be due to a prognostic rather than a predictive value. Therefore, we interrogated public databases and data available at our biobank to assess the frequency in CRC of variants in *MAP2K1*, *FBXW7*, and *NF1* and the correlation with clinical-pathological features. In particular, we investigated whether these variants are found in left-sided tumors, in which the use of anti-EGFR monoclonal antibodies is highly recommended, as well as their prognostic significance.

In the cBioPortal database, variants of the *MAP2K1* gene are reported at frequencies of 1.7% in CRC patients (Table 1) and correlated with worse disease/progression-free survival (Logrank Test P-Value: 1.815e-3), but not with overall survival. Although *MAP2K1* mutations were more frequent in right-sided tumors, they were also detected in left colon tumors (Table 1). *MAP2K1* variants were also more frequent among *KRAS/NRAS/BRAF* wt patients as compared with unselected CRC (Table 1). The *MAP2K1* mutations previously reported to be associated with de novo and acquired resistance to anti-EGFR MoAbs reside in the negative regulatory domain of the MEK1 protein and are associated with a gain of function of the protein [11,14]. We interrogated our database for these specific mutations. Among 939 CRC cases tested at our laboratory for *MAP2K1* mutations within clinical practice and clinical research, only 2 (0.2%) showed MEK1 gain of function variants (c.199G>A, p.Asp67Asn; c.169A>G, p.Lys57Glu), thus confirming the rarity of these specific genetic alterations in CRC. Importantly, both variants were found in patients with left-sided CRC.

Alterations in *NF-1* are described in 4.9% of CRC (Table 1) and do not correlate with survival, based on cBioPortal data. The frequency of these mutations is higher in right-sided tumors, without any significant difference between unselected CRC and *KRAS/NRAS/BRAF* wt carcinomas (Table 1).

Finally, we interrogated the cBioPortal database for the frequency of *FBXW7* variants in CRC. Mutations in this gene are described in 12.5% of CRC patients and do not show correlation with survival. The frequency of *FBXW7* variants is slightly higher in right-sided tumors (Table 1). These mutations also showed a slightly lower frequency in tumors that did not carry mutations in *KRAS*, *NRAS*, and *BRAF* genes (Table 1).

## 3. Discussion

Despite treatment with anti-EGFR MoAbs significantly improves the outcome of *KRAS/NRAS/BRAF* wt mCRC patients, additional mechanisms of resistance might limit their activity. In this respect, several previous reports hypothesized that different genetic alterations might play a role in the de novo resistance to anti-EGFR agents [7,9,10]. However, this study is the first to address this question in patients treated with first-line anti-EGFR MoAbs within a prospective clinical study. Although we could analyze a limited number of patients for which tumor samples were available, we identified variants in several genes that might be potentially involved in the resistance to anti-EGFR agents.

Among patients with the shorter PFS, we detected variants in genes that have been already associated with resistance to anti-EGFR MoAbs in CRC, such as *KRAS*, *MAP2K1*, and *FBXW7*, as well as in the new candidate gene *NF1*. We must acknowledge that the lack of a control arm prevents from the possibility to define whether these variants are predictive or prognostic. However, the mutations that we identified are mechanistically linked to EGFR-signaling, suggesting that they are good candidates as possible drivers of resistance to anti-EGFR agents. In addition, data from public database suggest that *FBXW7* and *NF1* mutations are not associated with worse prognosis in CRC.

We found that a patient with KRAS p.Gln61 mutation at a very low allelic frequency in the tumor tissue and a liquid biopsy positive for the same KRAS variant had a quite short PFS. Sub-clonal *KRAS* mutations have been reported to occur in CRC [20,21]. *KRAS* variants at low allelic frequency are unlikely to determine resistance to anti-EGFR MoAbs in mCRC patients [22]. However, we and other groups described that some *RAS* wt mCRC patients with clinical resistance to anti-EGFR agents had a liquid biopsy positive for *KRAS* mutations and carried the same variant at very low allelic frequency in the primary tumor [13,23]. Liquid biopsy is not expected to be positive for mutations present at very low allelic frequency in the tumor tissue due to the limit of sensitivity of this technique [24]. Therefore, we hypothesize that minor tumor clones carrying *KRAS* variants might be enriched during tumor progression in the metastatic sites, thus determining resistance to anti-EGFR MoAbs and positivity of liquid biopsy. These findings suggest that analysis of tumor tissue and liquid biopsy can provide complementary information on sensitivity to anti-EGFR agents in patients with metastatic disease.

Mutations in p.Lys57 of MEK1 have been previously found to be associated with de novo and acquired resistance to anti-EGFR agents [11,14]. Mechanistically, these variants lead to constitutive activation of MEK1 and increased downstream signaling. Although mutations in this site are quite rare in CRC, their identification might lead to a better identification of patients with primary resistance to anti-EGFR MoAbs. In addition, combinations of anti-EGFR MoAbs, BRAF, and MEK inhibitors, that are highly active in patients with *BRAF* mutations, might be effective also in patients carrying MAP2K1 activating variants [25].

The role of FBXW7 in the resistance to anti-EGFR MoAbs is more controversial. *FBXW7* is a tumor suppressor gene that encodes the substrate recognition component of SKP1–Cullin1–F-box protein ubiquitin E3 ligase complexes, which in turn negatively regulate the intracellular abundance of several key oncogenic proteins [26]. In particular, its loss of function leads to increased levels of total and activated mTOR. Variants in *FBXW7* have been described in 6% to 10% of CRC [27,28]. In patients with mCRC, *FBXW7* missense mutations are associated with *PIK3CA* mutations and with a shorter overall survival (OS) [28]. By using gene expression data, loss of FBXW7 was also correlated with a gene expression profile of RAS activation and resistance to anti-EGFR MoAbs in CRC [29]. In agreement with these data, *FBXW7* variants were identified in cases with resistance to anti-EGFR MoAbs in a retrospective study of 67 mCRC patients treated with different anti-EGFR MoAbs and chemotherapy regimens [30]. Interestingly, in this study the majority of the FBXW7 mutations correlating with patients’ outcome were in the WD40 domain, which is involved in FBXW7 binding to its substrates [16]. All the FBXW7 mutations identified in our study occurred in the WD40 domain. A *FBXW7* variant was found in our study in a patient with a good response to cetuximab-based therapy. The tumor of this patient carried also an amplification of *GAS6*, whose elevated expression has been previously associated with a favorable prognosis in CRC [31]. These findings suggest that the complexity of the genetic landscape of CRC rather than the single alteration might affect the outcome of CRC patients and their sensitivity to anti-EGFR agents.

Our data also point out to *NF1* variants as a possible new biomarker of resistance to EGFR MoAbs. *NF1* mutations are reported in 4.9% of *KRAS/NRAS/BRAF* wt CRC in the cBioPortal database (Table 1). Interestingly, both variants identified in our study have not been previously described, underlying the need to use technologies that cover the full-length gene for the analysis of *NF1* mutations. In a recent study of cetuximab-based therapy in a small cohort of Chinese mCRC patients (n. 33), the presence of *NF1* mutations was associated with the shortest PFS [32]. Importantly, reduced NF1 expression has been also demonstrated to confer resistance to EGFR inhibition in lung cancer [33]. Collectively, these preliminary observations suggest the possible role of NF1 in the resistance to anti-EGFR MoAbs in mCRC.

We observed that a patient with *ERBB2* copy number gain had a very good response to first line cetuximab-based therapy. This observation contrasts with findings suggesting a role of *ERBB2* gene amplification in the resistance to anti-EGFR MoAbs in patients with mCRC [34,35]. However, these studies assessed the correlation between *ERBB2* amplification and sensitivity to anti-EGFR MoAbs in patients receiving these drugs exclusively as second- or third-line treatment. Previous studies have shown activity of anti-EGFR agents in CRC patients with partial *ERBB2* amplification [36]. In addition, a recent editorial highlighted a good PFS in selected patients with *ERBB2* amplified tumors in the HERACLES trial [37]. Although our data deriving from a single patient cannot be generalized, our observation highlights the relevance to assess biomarkers in the first-line setting before making conclusions on their predictive role.

In 4 out of 10 tumors from patients with shorter PFS we were not able to detect genetic alterations that might be associated with resistance to anti-EGFR agents. A whole exome sequencing approach might be able to discover genetic alterations that are not covered by targeting sequencing panels. However, more complex mechanisms involving the interaction of CRC cells with tumor microenvironment might also play a relevant role in determining the sensitivity/resistance of CRC to anti-EGFR MoAbs [38].

Recent findings suggest that anti-EGFR MoAbs are highly active in patients with left-sided tumors, whereas they have little activity in right-sided tumors [6]. In agreement with this hypothesis, two out of three patients with right-sided tumors included in this analysis had a short PFS. Importantly, the possible mechanisms of resistance that we identified in this study were found in patients with left-sided tumors, who are usually treated with anti-EGFR agents in the clinical practice. Therefore, these biomarkers might help to define better the population of patients with high sensitivity to EGFR blockade.

Finally, patients with longer PFS in our series carried more CNVs as compared with patients with shorter PFS. This might be due to the different genetic background of these tumors. In fact, CNVs are more frequent in tumors related to the chromosomal instability pathway that have been suggested to be more sensitive to anti-EGFR agents [39].

## 4. Materials and Methods

### 4.1. Patients

The CAPRI-GOIM clinical trial is a nonprofit academic, open-label, multicenter study carried out by the GOIM cooperative group (EudraCT number: 2009-014041-81). Patients with *KRAS* exon 2 wild-type mCRC, as assessed by local pathology laboratories, received first-line treatment with FOLFIRI plus cetuximab until progression or unacceptable toxicity or patient refusal. After progression, patients were randomized (1:1) to receive FOLFOX or FOLFOX plus cetuximab as second line therapy [12,21]. The primary end point was PFS. Secondary end points included overall response rate (ORR), defined as the proportion of patients with confirmed complete responses (CR) plus partial responses (PR), and overall survival (OS). Responses were evaluated by local radiologist in each participating center according to RECIST criteria. Inclusion criteria were: age of 18 years or older, histologically confirmed adenocarcinoma of the colon or rectum, first occurrence of metastatic disease, Eastern Cooperative Oncology Group (ECOG) performance status score of 0 or 1, and adequate hematologic, hepatic, and renal function. Exclusion criteria were: previous exposure to an anti-EGFR therapy or to irinotecan-based chemotherapy, previous chemotherapy for metastatic colorectal cancer, or any investigational drug in the 30-day period before the start of treatment in the study. In the first-line treatment, about 600 patients were screened for KRAS mutations to identify 340 eligible patients. Patients were evaluated every 8 weeks to assess the response to treatment. The protocol was approved by the Ethical Committee of the Istituto Tumori Giovanni Paolo II Bari on July 23, 2009 (N. 303) and it was next approved in each center by local independent Ethics Committee.

The CAPRI-GOIM clinical trial is a nonprofit academic, open-label, multicenter study carried out by the GOIM cooperative group (EudraCT number: 2009-014041-81). Patients with *KRAS* exon 2 wild-type mCRC, as assessed by local pathology laboratories, received first-line treatment with FOLFIRI plus cetuximab until progression or unacceptable toxicity or patient refusal. After progression, patients were randomized (1:1) to receive FOLFOX or FOLFOX plus cetuximab as second line therapy [12,21]. The primary end point was PFS. Secondary end points included overall response rate (ORR), defined as the proportion of patients with confirmed complete responses (CR) plus partial responses (PR), and overall survival (OS). Responses were evaluated by local radiologist in each participating center according to RECIST criteria. Inclusion criteria were: age of 18 years or older, histologically confirmed adenocarcinoma of the colon or rectum, first occurrence of metastatic disease, Eastern Cooperative Oncology Group (ECOG) performance status score of 0 or 1, and adequate hematologic, hepatic, and renal function. Exclusion criteria were: previous exposure to an anti-EGFR therapy or to irinotecan-based chemotherapy, previous chemotherapy for metastatic colorectal cancer, or any investigational drug in the 30-day period before the start of treatment in the study. In the first-line treatment, about 600 patients were screened for KRAS mutations to identify 340 eligible patients. Patients were evaluated every 8 weeks to assess the response to treatment. The protocol was approved by the Ethical Committee of the Istituto Tumori Giovanni Paolo II Bari on July 23, 2009 (N. 303) and it was next approved in each center by local independent Ethics Committee.

Baseline CRC tumor samples (182/340, 53.5%) were retrospectively analyzed with a targeted sequencing panel covering hotspot mutations in 22 genes [12]. Tumor tissue for further genetic analysis was available for 21 *KRAS/NRAS/BRAF/PIK3CA* wt mCRC patients.

### 4.2. Targeted Sequencing

Formalin-fixed, paraffin-embedded (FFPE) tumor samples were obtained before first-line treatment with cetuximab-based therapy. Tumor tissues were analyzed with the Oncomine Comprehensive Assay v1 (Thermo Fisher Scientific, Milan, Italy) using the Ion Torrent semiconductor sequencing. Libraries were prepared starting from 10 ng of genomic DNA or RNA according to the manufacturer’s instructions. For each sample the DNA and RNA libraries were combined and clonally amplified on Ion sphere particles (ISPs) by emulsion PCR performed on the Ion One Touch 2 instrument with the Ion PGM template OT2 200 Kit (Thermo Fisher Scientific) according to the manufacturer’s instructions. Then, ISPs were enriched, loaded on an Ion 318 chip and sequenced on a PGM sequencer with the Ion PGM sequencing 200 kit v2 according to the manufacturer’s instructions. The raw data were analyzed using the Torrent Suite Software v5.0 (Thermo Fisher Scientific). Mutations were detected using the Ion Reporter Software v5.0 with low stringency settings. Each mutation was verified in the Integrative genome viewer (IGV) from the Broad Institute (http://www.broadinstitute.org/igv/). All genetic variants were confirmed by Sanger sequencing or Droplet Digital PCR. The QX200 Droplet Digital PCR (ddPCR) System (Bio-Rad, Milan, Italy) was used to perform digital PCR and data were analyzed using the QuantaSoft analytical software v1.7.4 (Bio-Rad, Milan, Italy).

## 5. Conclusions

Our data suggest that a wide genetic profiling of *KRAS/NRAS/BRAF/PIK3CA* wt mCRC patients might improve the ability to select patients who are highly sensitive to anti-EGFR MoAbs and provide the rationale for the development of therapeutic approaches with agents targeting different signaling pathways, alone or in combination with anti-EGFR drugs. Further studies are required to confirm the role of the identified genetic alterations as biomarkers predictive of response to anti-EGFR MoAbs.

## Figures and Tables

**Table 1:** Frequency of *MAP2K1*, *FBXW7*, and *NF1* mutations in CRC and correlation with sidedness based on cBioPortal database.

	Unselected CRC			*KRAS/NRAS/BRAF* Wild-Type CRC		
Genes	All	Left-Sided	Right-Sided	All	Left-Sided	Right-Sided
*MAP2K1*	1.7% (61/3473)	1.1% (10/878)	3.1% (13/416)	2.7% (27/1011)	1.6% (8/497)	6% (7/117)
*NF1*	4.9% (169/3473)	3.9% (34/878)	7.2% (30/416)	5% (51/1011)	4.2% (21/497)	7.7% (9/117)
*FBXW7*	12.5% (433/3473)	13.3% (117/878)	15.6% (65/416)	8.5% (86/1011)	10.3% (51/497)	10.3% (12/117)

## Supplementary Materials

The following are available online at https://www.mdpi.com/2072-6694/11/6/859/s1, Table S1: Clinical data, histopathological characteristics and variants identified in the cohort of 21 quadruple-wt mCRC patients.

## References

[1] Cremolini C., Schirripa M., Antoniotti C., Moretto R., Salvatore L., Masi G., Falcone A., Loupakis F. (2015). First-line chemotherapy for mCRC-a review and evidence-based algorithm. Nat. Rev. Clin. Oncol..

[2] Douillard J.Y., Oliner K.S., Siena S., Tabernero J., Burkes R., Barugel M., Humblet Y., Bodoky G., Cunningham D., Jassem J. (2013). Panitumumab-FOLFOX4 treatment and RAS mutations in colorectal cancer. N. Engl. J. Med..

[3] Van Cutsem E., Lenz H.J., Kohne C.H., Heinemann V., Tejpar S., Melezinek I., Beier F., Stroh C., Rougier P., van Krieken J.H. (2015). Fluorouracil, leucovorin, and irinotecan plus cetuximab treatment and RAS mutations in colorectal cancer. J. Clin. Oncol..

[4] Cremolini C., Pietrantonio F. (2016). How the lab is changing our view of colorectal cancer. Tumori.

[5] Normanno N., Tejpar S., Morgillo F., De Luca A., Van Cutsem E., Ciardiello F. (2009). Implications for KRAS status and EGFR-targeted therapies in metastatic CRC. Nat. Rev. Clin. Oncol..

[6] Arnold D., Lueza B., Douillard J.Y., Peeters M., Lenz H.J., Venook A., Heinemann V., Van Cutsem E., Pignon J.P., Tabernero J. (2017). Prognostic and predictive value of primary tumour side in patients with RAS wild-type metastatic colorectal cancer treated with chemotherapy and EGFR directed antibodies in six randomized trials. Ann. Oncol..

[7] Bertotti A., Migliardi G., Galimi F., Sassi F., Torti D., Isella C., Cora D., Di Nicolantonio F., Buscarino M., Petti C. (2011). A molecularly annotated platform of patient-derived xenografts (“xenopatients”) identifies HER2 as an effective therapeutic target in cetuximab-resistant colorectal cancer. Cancer Discov..

[8] Cremolini C., Morano F., Moretto R., Berenato R., Tamborini E., Perrone F., Rossini D., Gloghini A., Busico A., Zucchelli G. (2017). Negative hyper-selection of metastatic colorectal cancer patients for anti-EGFR monoclonal antibodies: The PRESSING case-control study. Ann. Oncol..

[9] De Roock W., De Vriendt V., Normanno N., Ciardiello F., Tejpar S. (2011). KRAS, BRAF, PIK3CA, and PTEN mutations: Implications for targeted therapies in metastatic colorectal cancer. Lancet Oncol..

[10] Pietrantonio F., Di Nicolantonio F., Schrock A.B., Lee J., Tejpar S., Sartore-Bianchi A., Hechtman J.F., Christiansen J., Novara L., Tebbutt N. (2017). ALK, ROS1, and NTRK Rearrangements in Metastatic Colorectal Cancer. J. Natl. Cancer Inst..

[11] Russo M., Siravegna G., Blaszkowsky L.S., Corti G., Crisafulli G., Ahronian L.G., Mussolin B., Kwak E.L., Buscarino M., Lazzari L. (2016). Tumor Heterogeneity and Lesion-Specific Response to Targeted Therapy in Colorectal Cancer. Cancer Discov..

[12] Ciardiello F., Normanno N., Maiello E., Martinelli E., Troiani T., Pisconti S., Giuliani F., Barone C., Carteni G., Rachiglio A.M. (2014). Clinical activity of FOLFIRI plus cetuximab according to extended gene mutation status by next-generation sequencing: Findings from the CAPRI-GOIM trial. Ann. Oncol..

[13] Normanno N., Esposito Abate R., Lambiase M., Forgione L., Cardone C., Iannaccone A., Sacco A., Rachiglio A.M., Martinelli E., Rizzi D. (2018). RAS testing of liquid biopsy correlates with the outcome of metastatic colorectal cancer patients treated with first-line FOLFIRI plus cetuximab in the CAPRI-GOIM trial. Ann. Oncol..

[14] Bertotti A., Papp E., Jones S., Adleff V., Anagnostou V., Lupo B., Sausen M., Phallen J., Hruban C.A., Tokheim C. (2015). The genomic landscape of response to EGFR blockade in colorectal cancer. Nature.

[15] Marks J.L., Gong Y., Chitale D., Golas B., McLellan M.D., Kasai Y., Ding L., Mardis E.R., Wilson R.K., Solit D. (2008). Novel MEK1 mutation identified by mutational analysis of epidermal growth factor receptor signaling pathway genes in lung adenocarcinoma. Cancer Res..

[16] Yeh C.H., Bellon M., Nicot C. (2018). FBXW7: A critical tumor suppressor of human cancers. Mol. Cancer.

[17] Laurent-Puig P., Cayre A., Manceau G., Buc E., Bachet J.B., Lecomte T., Rougier P., Lievre A., Landi B., Boige V. (2009). Analysis of PTEN, BRAF, and EGFR status in determining benefit from cetuximab therapy in wild-type KRAS metastatic colon cancer. J. Clin. Oncol..

[18] Loupakis F., Pollina L., Stasi I., Ruzzo A., Scartozzi M., Santini D., Masi G., Graziano F., Cremolini C., Rulli E. (2009). PTEN expression and KRAS mutations on primary tumors and metastases in the prediction of benefit from cetuximab plus irinotecan for patients with metastatic colorectal cancer. J. Clin. Oncol..

[19] Sartore-Bianchi A., Martini M., Molinari F., Veronese S., Nichelatti M., Artale S., Di Nicolantonio F., Saletti P., De Dosso S., Mazzucchelli L. (2009). PIK3CA mutations in colorectal cancer are associated with clinical resistance to EGFR-targeted monoclonal antibodies. Cancer Res..

[20] McGranahan N., Favero F., de Bruin E.C., Birkbak N.J., Szallasi Z., Swanton C. (2015). Clonal status of actionable driver events and the timing of mutational processes in cancer evolution. Sci. Transl. Med..

[21] Normanno N., Rachiglio A.M., Lambiase M., Martinelli E., Fenizia F., Esposito C., Roma C., Troiani T., Rizzi D., Tatangelo F. (2015). Heterogeneity of KRAS, NRAS, BRAF and PIK3CA mutations in metastatic colorectal cancer and potential effects on therapy in the CAPRI GOIM trial. Ann. Oncol..

[22] Laurent-Puig P., Pekin D., Normand C., Kotsopoulos S.K., Nizard P., Perez-Toralla K., Rowell R., Olson J., Srinivasan P., Le Corre D. (2015). Clinical relevance of KRAS-mutated subclones detected with picodroplet digital PCR in advanced colorectal cancer treated with anti-EGFR therapy. Clin. Cancer Res..

[23] Khan K.H., Cunningham D., Werner B., Vlachogiannis G., Spiteri I., Heide T., Mateos J.F., Vatsiou A., Lampis A., Damavandi M.D. (2018). Longitudinal Liquid Biopsy and Mathematical Modeling of Clonal Evolution Forecast Time to Treatment Failure in the PROSPECT-C Phase II Colorectal Cancer Clinical Trial. Cancer Discov..

[24] Normanno N., Cervantes A., Ciardiello F., De Luca A., Pinto C. (2018). The liquid biopsy in the management of colorectal cancer patients: Current applications and future scenarios. Cancer Treat. Rev..

[25] Van Cutsem E., Huijberts S., Grothey A., Yaeger R., Cuyle P.J., Elez E., Fakih M., Montagut C., Peeters M., Yoshino T. (2019). Binimetinib, Encorafenib, and Cetuximab Triplet Therapy for Patients With BRAF V600E-Mutant Metastatic Colorectal Cancer: Safety Lead-In Results From the Phase III BEACON Colorectal Cancer Study. J. Clin. Oncol..

[26] Mao J.H., Kim I.J., Wu D., Climent J., Kang H.C., DelRosario R., Balmain A. (2008). FBXW7 targets mTOR for degradation and cooperates with PTEN in tumor suppression. Science.

[27] Adua D., Di Fabio F., Ercolani G., Fiorentino M., Gruppioni E., Altimari A., Rojas Limpe F.L., Normanno N., Pinna A.D., Pinto C. (2017). Heterogeneity in the colorectal primary tumor and the synchronous resected liver metastases prior to and after treatment with an anti-EGFR monoclonal antibody. Mol. Clin. Oncol..

[28] Korphaisarn K., Morris V.K., Overman M.J., Fogelman D.R., Kee B.K., Raghav K.P.S., Manuel S., Shureiqi I., Wolff R.A., Eng C. (2017). FBXW7 missense mutation: A novel negative prognostic factor in metastatic colorectal adenocarcinoma. Oncotarget.

[29] Guinney J., Ferte C., Dry J., McEwen R., Manceau G., Kao K.J., Chang K.M., Bendtsen C., Hudson K., Huang E. (2014). Modeling RAS phenotype in colorectal cancer uncovers novel molecular traits of RAS dependency and improves prediction of response to targeted agents in patients. Clin. Cancer Res..

[30] Lupini L., Bassi C., Mlcochova J., Musa G., Russo M., Vychytilova-Faltejskova P., Svoboda M., Sabbioni S., Nemecek R., Slaby O. (2015). Prediction of response to anti-EGFR antibody-based therapies by multigene sequencing in colorectal cancer patients. BMC Cancer.

[31] Akitake-Kawano R., Seno H., Nakatsuji M., Kimura Y., Nakanishi Y., Yoshioka T., Kanda K., Kawada M., Kawada K., Sakai Y. (2013). Inhibitory role of Gas6 in intestinal tumorigenesis. Carcinogenesis.

[32] Mei Z., Shao Y.W., Lin P., Cai X., Wang B., Ding Y., Ma X., Wu X., Xia Y., Zhu D. (2018). SMAD4 and NF1 mutations as potential biomarkers for poor prognosis to cetuximab-based therapy in Chinese metastatic colorectal cancer patients. BMC Cancer.

[33] De Bruin E.C., Cowell C., Warne P.H., Jiang M., Saunders R.E., Melnick M.A., Gettinger S., Walther Z., Wurtz A., Heynen G.J. (2014). Reduced NF1 expression confers resistance to EGFR inhibition in lung cancer. Cancer Discov..

[34] Raghav K., Loree J.M., Morris J.S., Overman M.J., Yu R., Meric-Bernstam F., Menter D., Korphaisarn K., Kee B., Muranyi A. (2019). Validation of HER2 Amplification as a Predictive Biomarker for Anti–Epidermal Growth Factor Receptor Antibody Therapy in Metastatic Colorectal Cancer. JCO Precis. Oncol.ogy..

[35] Sartore-Bianchi A., Amatu A., Porcu L., Ghezzi S., Lonardi S., Leone F., Bergamo F., Fenocchio E., Martinelli E., Borelli B. (2019). HER2 Positivity Predicts Unresponsiveness to EGFR-Targeted Treatment in Metastatic Colorectal Cancer. Oncologist.

[36] Martin V., Landi L., Molinari F., Fountzilas G., Geva R., Riva A., Saletti P., De Dosso S., Spitale A., Tejpar S. (2013). HER2 gene copy number status may influence clinical efficacy to anti-EGFR monoclonal antibodies in metastatic colorectal cancer patients. Br. J. Cancer.

[37] Bregni G., Sciallero S., Sobrero A. (2019). HER2 Amplification and Anti-EGFR Sensitivity in Advanced Colorectal Cancer. JAMA Oncol..

[38] Dienstmann R., Vermeulen L., Guinney J., Kopetz S., Tejpar S., Tabernero J. (2017). Consensus molecular subtypes and the evolution of precision medicine in colorectal cancer. Nat. Rev. Cancer.

[39] Sveen A., Bruun J., Eide P.W., Eilertsen I.A., Ramirez L., Murumagi A., Arjama M., Danielsen S.A., Kryeziu K., Elez E. (2018). Colorectal Cancer Consensus Molecular Subtypes Translated to Preclinical Models Uncover Potentially Targetable Cancer Cell Dependencies. Clin. Cancer Res..

