# Supplementary material for: Genomic Profiling of KRAS/NRAS/BRAF/PIK3CA Wild-Type Metastatic Colorectal Cancer Patients Reveals Novel Mutations in Genes Potentially Associated with Resistance to Anti-EGFR Agents
Source: Cancers (Basel). 2019 Jun 20;11(6):859. doi: 10.3390/cancers11060859 (PMC6627713; doi:10.3390/cancers11060859)
Supplement: Supplementary file 1 [file cancers-11-00859-s001.pdf]

Supplementary Table 1: Clinical data, histopathological characteristics and variants identified in the cohort of 21 quadruple-wt mCRC patients.

ID	Gender	Age	Race	Site of primary tumor	Histological grading	Tumor Stage	Primary tumor resected	Metastatic sites (n°)	Location of progression	SNVs and Indels	CNVs : fold change
**P1**	Female	35	Caucasian	Colon (left)	G2	4	Yes	1	Liver	APC: p.Gln1291Ter (c.3871C>T)<br>TP53: p.Arg273His (c.818G>A)	–
**P2**	Male	54	Caucasian	Colon (left)	G2	4	Yes	1	Liver	APC: p.Ser1400fs (c.4199_4199delC)<br>TP53: p.Gly245Cys (c.733G>T)	–
**P3**	Female	64	Caucasian	Colon (left)	G3	4	Yes	3	Liver, Lung, Lymph node	MAP2K1: p.Lys57Glu (c.169A>G)<br>TP53: p.Arg175His (c.524G>A)	–
**P4**	Female	61	Caucasian	Colon (left)	G2	3	Yes	1	Lymph node	APC: p.Arg876Ter (c.2626C>T)<br>TP53: p.Leu93fs (c.275_276ins)<br>FBXW7: p.Gly423Val (c.1268G>T)	GAS6: 5.59
**P5**	Male	72	Caucasian	Rectum	G1	4	Yes	1	Liver	APC: p.Arg216Ter (c.646C>T); p.Glu1353Ter (c.4057G>T)<br>TP53: p.Gly266Arg (c.796G>A); p.Val157Phe (c.469G>T)	–
**P6**	Male	77	Caucasian	Colon (left)	G2	4	No	1	Liver	APC: p.Arg554Ter (c.1660C>T)<br>TP53: p.Arg175His (c.524G>A)	GAS6: 5.45<br>APC: 0.45<br>PIK3R1: 0.48<br>BCL2L1: 6.3
**P7**	Female	51	Caucasian	Rectum	G2	4	Yes	1	Liver	KRAS: p.Gln61His (c.183A>T)	–
**P8**	Male	69	Caucasian	Colon (left)	G2	3	Yes	2	Liver	APC: p.Lys1030Ter (c.3088A>T); p.Thr1493fs (c.4467_4468insCATTTTG)<br>PTPN11: p.Glu39Lys (c.205G>A)<br>TP53: p.Arg248Gln (c.743G>A)	MYC: 5.11<br>ZNF217: 4.7<br>BCL2L1: 4.67
**P9**	Male	69	Caucasian	Colon (left)	G2	4	Yes	1	Lymph node	TP53: p.Met273Ile (c.711G>A)	–
**P10**	Male	70	Caucasian	Colon (left)	G2	4	Yes	2	Bone	APC: p.Ser1356Ter (c.4067C>A)<br>TP53: p.Pro27fs (c.80_80delC)	BCL2L1: 4.96
**P11**	Male	56	Caucasian	Colon (left)	G2	4	Yes	1	Liver	TP53: p.Arg213Ter; (c.637C>T)	–
**P12**	Female	64	Caucasian	Colon (left)	G2	4	Yes	1	Liver	APC: p.Gln1367Ter (c.4098_4099delTCinsAT)<br>ATM: p.Val410Ala (c.1229T>C)<br>TP53: p.Cys229fs (c.686_687_80delGT)	MYC: 5.31
**P13**	Female	50	Caucasian	Colon (right)	G3	4	Yes	2	Lung, Lymph node	APC: p.Ser1355fs (c.4060_4064delTTTTC)<br>TP53: p.Val173Met (c.517G>A)	–

**P14**	Female	57	Caucasian	Rectum	G2	3	Yes	3	Liver, Lung, Lymph node	FBXW7: p.Asp600Asn (c.1798G>A)<br>APC: p.Gln1090Ter (c.3268C>T); p.Glu1353Ter (c.4057G>T)<br>TP53: p.Cys176Phe (c.527G>T)	TP53: 0.54
**P15**	Male	68	Caucasian	Colon (left)	G2	4	Yes	2	Liver, Bone	CTNNB1: p.Ser45Phe (c.134C>T)<br>TP53: p.Phe54fs (c.162_162delC)	GAS6: 8.25<br>APC: 0.57<br>ZNF217: 8.2<br>BCL2L1: 8.05<br>FLT3: 7
**P16**	Male	58	Caucasian	Colon (right)	G1	4	Yes	1	Liver	APC: p.Ala199fs (c.589_590insGAGTT); p.Gln1367Ter (c.4099C>T)<br>TP53: p.Cys275fs (c.823_824insT): p.Arg248Trp (c.742C>T); p.Ast186Val (c.557A>G)	ERBB2: 78.99
**P17**	Male	72	Caucasian	Rectum	G2	4	Yes	2	Not available	APC: p.Ser1355fs (c.4060_4061delTT)<br>PIK3R1: p.Pro568fs (c.1698_1699InsA)<br>CDKN2A: p.Val25fs (c.73_74InsG)<br>PTEN: p.Glu235fs (c.700_701InsG)<br>TP53: p.Arg280fs (c.837_838InsG)	APEX1: 5.47
**P18**	Male	72	Caucasian	Colon (left)	G3	Not available	Yes	2	Liver	FBXW7: p.Arg505Cys (c.1513C>T)<br>APC: p.Val1472fs (c.4415_4415delT)<br>TP53: p.Arg248Trp (c.742C>T)	–
**P19**	Female	62	Caucasian	Colon (right)	G2	2	Yes	3	Liver, Lung, Peritoneum	APC: p.Ile1580fs (c.4733_4734insTA)<br>TP53: p.Arg175His (c.524G>A)	–
**P20**	Male	60	Caucasian	Rectum	G2	4	Yes	2	Liver, Lung	NF1: p.Asn214fs (c.638_639insA)	–
**P21**	Female	75	Caucasian	Colon (left)	G2	4	Yes	2	Liver	APC: p.Lys1310fs (c.3926_3927delAA)<br>TP53: p.Arg273Cys (c.817C>T)<br>NF1: p.Lys1701Ter (c.5101A>T)	GAS6: 6.04

SNVs: single nucleotide variants; Indels: insertions/deletions; CNVs: copy number variations
